# EVI1 as a Prognostic and Predictive Biomarker of Clear Cell Renal Cell Carcinoma

**DOI:** 10.3390/cancers12020300

**Published:** 2020-01-28

**Authors:** Luis Palomero, Lubomir Bodnar, Francesca Mateo, Carmen Herranz-Ors, Roderic Espín, Mar García-Varelo, Marzena Jesiotr, Gorka Ruiz de Garibay, Oriol Casanovas, José I. López, Miquel Angel Pujana

**Affiliations:** 1 ProCURE, Catalan Institute of Oncology (ICO), Bellvitge Institute for Biomedical Research (IDIBELL), L’Hospitalet del Llobregat, Barcelona 08908, Catalonia, Spain; lpalomero@iconcologia.net (L.P.); fmateo@iconcologia.net (F.M.); cherranz@iconcologia.net (C.H.-O.); rodericespin@gmail.com (R.E.); mgarciavaler@uoc.edu (M.G.-V.); grponce@iconcologia.net (G.R.d.G.); ocasanovas@iconcologia.net (O.C.); 2 Department of Oncology and Immunooncology, Hospital Ministry of the Interior and Administration with Warmia and Mazury Oncology Center, Olsztyn 10-719, Poland; 3 Department of Oncology, University of Warmia and Masuria, Olsztyn 10-719, Poland; 4 Department of Pathology, Military Institute of Medicine, Warsaw 04-141, Poland; marzena@obta.uw.edu.pl; 5 Department of Pathology, Cruces University Hospital, Biocruces Institute, Barakaldo 48903, Spain

**Keywords:** everolimus, EVI1, genetic association, mTOR, clear cell renal cell carcinoma

## Abstract

The transcription factor EVI1 plays an oncogenic role in several types of neoplasms by promoting aggressive cancer features. EVI1 contributes to epigenetic regulation and transcriptional control, and its overexpression has been associated with enhanced PI3K-AKT-mTOR signaling in some settings. These observations raise the possibility that EVI1 influences the prognosis and everolimus-based therapy outcome of clear cell renal cell carcinoma (ccRCC). Here, gene expression and protein immunohistochemical studies of ccRCC show that EVI1 overexpression is associated with advanced disease features and with poorer outcome—particularly in the CC-e.3 subtype defined by The Cancer Genome Atlas. Overexpression of an oncogenic EVI1 isoform in RCC cell lines confers substantial resistance to everolimus. The *EVI1* rs1344555 genetic variant is associated with poorer survival and greater progression of metastatic ccRCC patients treated with everolimus. This study leads us to propose that evaluation of EVI1 protein or gene expression, and of *EVI1* genetic variants may help improve estimates of prognosis and the benefit of everolimus-based therapy in ccRCC.

## 1. Introduction

The ecotropic viral integration site 1 gene (*Evi1*) locus was initially highlighted in mouse studies as a common retroviral integration genomic location causing myeloid tumors [1]. It encodes a dual-domain zinc-finger transcription factor with a fundamental role in regulating hematopoietic stem cell renewal and myeloid progenitor cell differentiation [2]. Abnormal overexpression of EVI1 and, therefore, activation of its underlying transcriptional program, are involved in up to a quarter of pediatric acute myeloid leukemia (AML), and influence prognosis and response to chemotherapy in this setting [3,4]. More recently, an oncogenic role for EVI1 has been broadened to include several epithelial cancers, similarly associated with aggressive features [5,6,7,8,9,10,11,12,13]. Analyses of genomic alterations across cancers have shown that chromosome 3q26.2, which harbors *EVI1*, is amplified in a variety of epithelial cancer types [14]. The specific locus is also known as the *MDS1* and *EVI1* complex locus (*MECOM*), and different isoforms may emerge with apparently opposite roles in tumorigenesis [5,15].

The oncogenic function of EVI1 is mediated by its established role on epigenetic regulation and transcriptional control [16,17,18]. EVI1 interacts with Polycomb-group (PcG) proteins to repress the expression of the tumor suppressor gene *PTEN* [19]. In parallel, it interacts with DNA methyltransferases causing a hypermethylation genomic signature [20]. In addition, EVI1 promotes specific gene silencing though interactions with histone methyltransferases [21,22,23]. As consequence of these functional associations, several key signaling pathways are altered promoting cancer. EVI1 negatively regulates TGF-β signaling through repression of *SMAD3* [24,25]. Furthermore, oncogenic EVI1 frequently enhances PI3K-AKT-mTOR signaling, as by repression of *PTEN* in leukemogenesis [19]. In intestinal epithelial cells, oncogenic EVI1 overactivates PI3K-AKT signaling in response to TGFβ-mediated and taxol-mediated apoptosis [6]. In breast cancer, overexpression of EVI1 is associated with poor prognosis [8], stem cell-like and lung-metastatic features, and resistance to allosteric mTOR inhibition [7]. Cancer stem cell-like and metastatic cells rely on enhanced mTOR activity, and EVI1 maintains this signaling by transcriptional upregulation of key pathway components and metastatic mediators [7].

The depicted associations between oncogenic EVI1 and abnormally enhanced mTOR activity raise the possibility that EVI1 influences cancer prognosis and therapeutic response in a clinical setting where this kinase plays a central role, that of ccRCC. This is the most frequent type of kidney cancer in adults, which is commonly caused by genetic alterations that hamper proper cellular response to hypoxia and, in turn, demand enhanced mTOR signaling [26,27]. Thus, everolimus, an allosteric mTOR inhibitor has been approved for the treatment of advanced ccRCC [28]. On the basis of these observations, we evaluated genetic variants and expression features of *EVI1*/EVI1 for their associations with ccRCC prognosis and therapeutic response. Our findings have the potential to improve estimates of ccRCC prognosis and the clinical benefit from everolimus.

## 2. Results

### 2.1. EVI1 Overexpression Is Associated with Progression Features and Poor Prognosis of ccRCC

**Figure 1:**
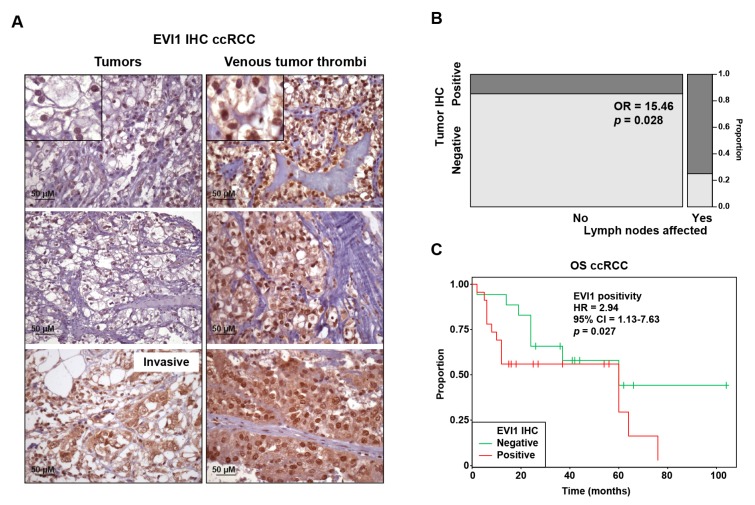
EVI1 expression in ccRCC tumors and venous tumor thrombi are associated with features of disease progression and poorer patient outcome. (**A**) Representative images of immunohistochemical detection of EVI1 in primary tumors (left panels) and venous tumor thrombi (right panels) from the cohort of 39 ccRCC cases (Supplementary Table S1). The top panel insets include magnified images showing nuclear positivity in cancer cells; weaker staining in the cytoplasm is also appreciated in some cases, which is consistent with observations in other cancer types [8,13]. (**B**) Grid showing the proportions of EVI1 IHC positivity in tumors relative to lymph node status in the same cohort. The odds ratio (OR) and corresponding *p*-value (Fisher’s exact test) for the association between EVI1 positivity and cancer-affected lymph node are shown. (**C**) Kaplan–Meier curves showing the association between EVI1 positivity and poorer survival in the same cohort. The multivariate (including age and gender) Cox regression overall survival (OS) hazard ratio (HR) estimate, 95% CI, and *p*-value are shown. The estimations for age and gender (male as reference) in this model were, respectively: HR = 1.05, 95% CI = 0.99–1.10, *p* = 0.07; and HR = 0.20, 95% CI = 0.07–0.54, *p* = 0.002.

In addition to myeloid leukemia, overexpression of EVI1 has been associated with aggressive phenotypes of breast cancer, colorectal, lung, ovarian, pancreatic, and prostate cancer [5,6,7,8,9,10,11,12,13]. To determine whether there is a similar link with ccRCC, EVI1-targeted immunohistochemistry assays were performed that included cases with tumor extension to the venous system (i.e., formation of venous tumor thrombus), since this is a feature of locally advanced disease [29]. Of 39 cases studied (Supplementary Table S1), 8 (20%) tumors and 18 (46%) venous tumor thrombi were found to be positive for EVI1 expression (Figure 1A). EVI1 was also found to be expressed in tumor-invasive areas of fat tissue (Figure 1A).

Analyses of histopathological data revealed a positive association between EVI1 expression and the presence of cancer-affected lymph nodes: odds ratio (OR) = 15.46, 95% confidence interval (CI) = 1.02-936.43, Fisher’s exact test *p* = 0.028 (Figure 1B). Combined analysis of the immunohistochemistry results from the tumors and venous tumor thrombi showed a significant association between EVI1 positivity and poorer patient outcome: multivariate Cox regression (including age and gender) overall survival (OS) EVI1 positivity hazard ratio (HR) = 2.94, 95% CI = 1.13–7.63, *p* = 0.027 (Figure 1C). These data suggest that EVI1 overexpression also contributes to the aggressiveness of ccRCC.

### 2.2. EVI1 Overexpression Confers ccRCC Cell Resistance to Everolimus

**Figure 2:**
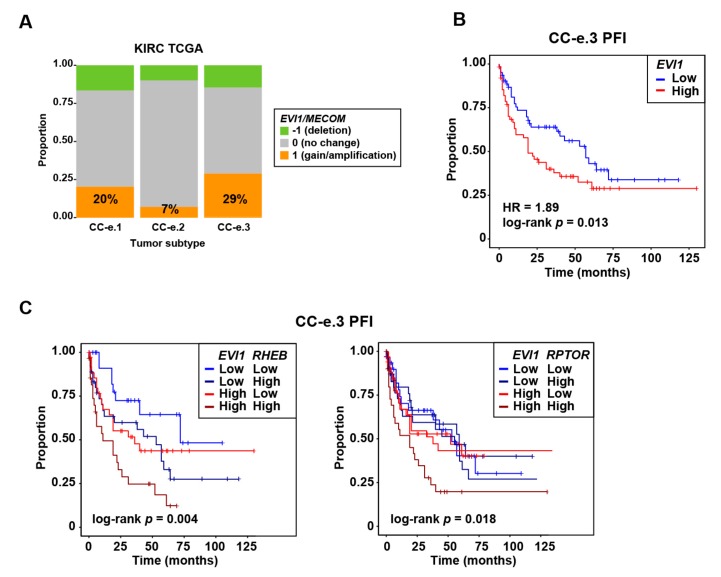
Frequent chromosome 3q26 *EVI1/MECOM* gain in the CC-e.3 KIRC/ccRCC subtype, gene expression association with poorer outcome in this subtype, and with *RHEB* and *RPTOR* influencing progression. (**A**) Graph showing the proportions of *EVI1/MECOM* genomic alterations (as depicted in the inset) in TCGA KIRC primary tumor subtypes (CC-e.1-3). The percentage of tumors with genomic gain in each subtype is shown. (**B**) Kaplan–Meier curves showing the association between *EVI1* overexpression and poorer PFI in the TCGA KIRC CC-e.3 cohort. This set was divided in two groups using the average expression value of *EVI1* as threshold (low or high *EVI1* tumor expression, being normally distributed). The multivariate (including age, gender, and tumor stage (I-II and III-IV) Cox regression HR estimate, 95% CI, and log-rank *p*-value are shown. (**C**) Kaplan–Meier curves showing the association between overexpression of *EVI1* and *RHEB* (left panel) or *RPTOR* (right panel) with poorer PFI in the TCGA KIRC CC-e.3 cohort. This set was divided in four groups using the average expression value of *EVI1* and *RHEB* or *RPTOR* as thresholds (low or high *EVI1* and low or high *RHEB*/*RPTOR* tumor expression). The log-rank *p*-values are shown.

Somatic gain of the 3q26 genomic region including *EVI1* was noted in the original study of ccRCC of The Cancer Genome Atlas (TCGA KIRC) [30]. Analysis of TCGA data identified the CC-e.3 as the ccRCC subtype with the greater proportion of tumors showing *EVI1* locus gain (Figure 2A). A high level of expression of EVI1 in this subtype—but not in the other KIRC subtypes (CC-e.1-2) and complete cohort—was found to be significantly associated with poorer outcome, as measured by a multivariate (including age, gender, and tumor stage) Cox regression analysis of progression-free interval (PFI; Figure 2B). The CC-e.3 subtype was identified by TCGA as the subgroup with a higher relative level of expression of markers of the epithelial–mesenchymal transition [30], which is consistent with the functional associations of EVI1 described in some cancer settings [6,7,15]. Indeed, *EVI1* expression in CC-e.3 tumors was found to be positively co-expressed with several metastasis-, invasion- and integrin-related curated gene sets (Supplementary Table S2). We previously identified the mTOR pathway components RHEB and RPTOR as being positively regulated by EVI1 in metastatic breast cancer with stem cell-like features [7]. Next, PFI analyses that took into account the expression of *EVI1* and either of these mTOR pathway components showed that outcome was significantly poorer when both genes were overexpressed (Figure 2C). Therefore, over-expression of *EVI1* may contribute to progression of certain ccRCC tumors.

**Figure 3:**
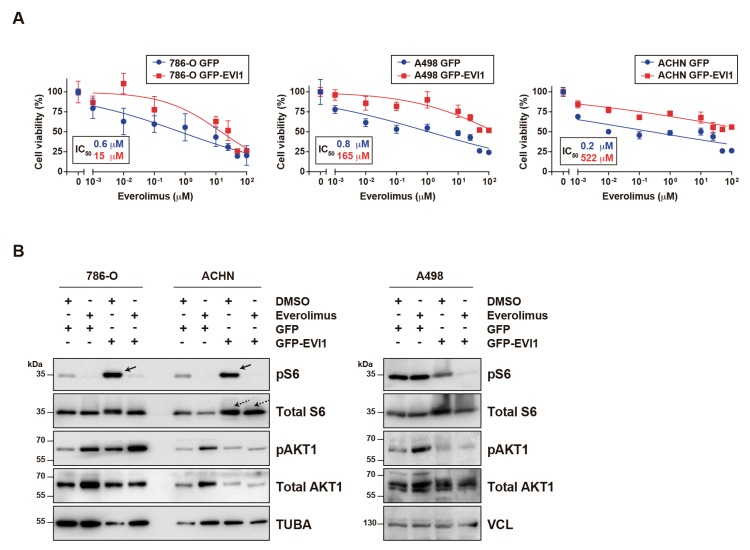
Ectopic oncogenic *EVI1* overexpression confers resistance to everolimus. (**A**) Graphs showing viability of RCC cells (Y-axis) transiently transfected and selected with GFP or GFP-EVI1^Del190–515^ expression constructs, and exposed to different concentrations of everolimus for 72 hours (X-axis). The two cell line conditions (GFP and GFP-EVI1^Del190–515^) are indicated in the insets and the estimated IC_50_ values are shown in the graphs. Each measure shows the mean and standard deviation of quintuplicate values. The curve fitting regression was computed using the log value versus normalized response. (**B**) Western blot results from the three RCC cell lines and two conditions, treated with DMSO or everolimus (20 nM), and analyzed for the levels of total S6 and pS6, total AKT1 and pAKT1, and loading control (tubulin, TUBA; or vinculin, VCL). The solid arrows (top left panel) indicate increased levels of pS6 in GFP-EVI1^Del190–515^ over-expressing 786-O and ACHN cells. The dashed arrows indicate increased levels of total S6 in GFP-EVI1^Del190–515^ over-expressing ACHN cells. Molecular weight markers are shown on the left side and expressed in kiloDalton (kDa).

Following on from the above observations, the responses of three RCC cell lines (ccRCC: 786-O and A498; and papillary RCC: ACHN) to everolimus upon ectopic overexpression of full-length EVI1 or EVI1^Del190–515^—two isoforms identified as oncogenic in ovarian cancer [15]—were assessed. The ACHN cell line was included because advanced papillary RCC may also be treated with everolimus [31]. While the full-length isoform did not show any significant effects, all three cell lines were considerably less sensitive to everolimus when GFP-EVI1^Del190–515^ was overexpressed relative to GFP alone, with >25-fold differences in the half-maximal inhibitory concentration (IC_50_) observed (Figure 3A). Rapalogs are primarily cytostatic instead of cytotoxic [32] and, at the highest everolimus concentration (100 µM) tested for 72 hours, the percentages of cell viability were for the GFP alone and GFP-EVI1^Del190–515^ conditions, respectively: 786-O, 26% and 21%; A498, 24% and 52%; and ACHN, 26% and 60%. 

Molecular analyses showed that overexpression of GFP-EVI1^Del190–515^ causes a robust increase of basal phospho-Ser235/236-ribosomal S6 protein (pS6) in two of the cell lines (786-O and ACHN; Figure 3B, left panels). In addition, an increase of total S6 was noted in ACHN cells (Figure 3B, left panels); however, no consistent changes were observed in the A498 assays (Figure 3B, right panels). Therefore, oncogenic EVI1 may be linked to enhanced mTOR signaling in some RCC cell models, which in turn might influence sensitivity to everolimus.

### 2.3. Common Genetic Variants in EVI1 Are Associated with Response to Everolimus of Metastatic ccRCC

The *EVI1* locus may be pleiotropic since at least 56 human traits have been linked to the corresponding genomic region (±50 kilobases of the *EVI1*/*MECOM* locus) in the results from diverse genome-wide association studies (Genome Browser GWAS catalog data, version GRCh37/hg19). Some of the identified traits might in turn be linked to known EVI1 functions. Variant forms at this locus have been associated with lung [33] and kidney function [34,35], and cancer risk, including breast and lung cancer susceptibility [36]. Therefore, we analyzed leading variants from these studies (rs1344555, rs16853722, and rs75316749, respectively) to establish their association with progression of metastatic ccRCC treated with everolimus (Supplementary Table S3).

**Figure 4:**
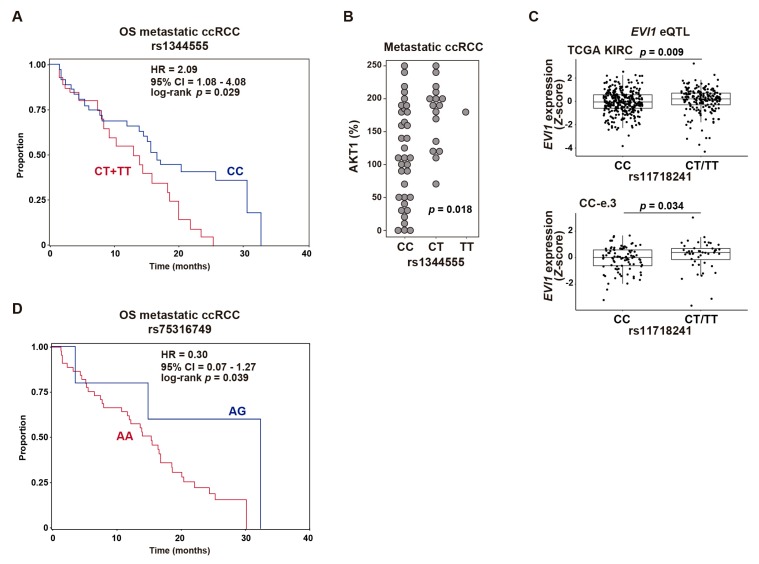
Common *EVI1* genetic variation is associated with response to everolimus of metastatic ccRCC. (**A**) Kaplan–Meier curves of OS based on rs1344555 C/C (*n* = 35) against C/T (*n* = 16) plus T/T (*n* = 1) genotypes. The univariate Cox regression HR estimate, 95% CI, and log-rank *p* are shown. (**B**) Graph showing the association between rs1344555 genotypes and AKT1 expression in metastatic RCC. The Kruskal–Wallis test *p*-value is shown. (**C**) Box plots showing the *EVI1* eQTL at rs11718241 in primary tumors from complete TCGA KIRC cohort (top panel) and from the CC-e.3 cohort (bottom panel). The Wilcoxon test *p*-values are shown. (**D**) Kaplan–Meier curves of OS of metastatic ccRCC based on rs75316749 A/G (*n* = 5) against A/A (*n* = 45) genotypes.

The rs1344555 variant was significantly associated with progression-free survival (PFS) and OS of everolimus-treated metastatic ccRCC: CC versus CT/TT genotypes PFS, hazard ratio (HR) = 1.96, 95% CI = 1.01–3.81, *p* = 0.047; and OS HR = 2.09, 95% CI = 1.08–4.08, *p =* 0.029 (Figure 4A). In this setting, the T allele was associated with a higher probability of disease progression and patient death and, in the original lung study, was associated with inferior organ function [33]. A significant association between the genotypes of rs1344555 and the percentage of AKT1-positive metastatic ccRCC cells was revealed (Kruskal–Wallis test *p* = 0.018; Figure 4B). Notably, a TCGA-genotyped variant correlated with rs1344555, rs11718241 (*r^2^* = 0.94 in European populations), proved to be an expression quantitative locus (eQTL) for *EVI1* in the total KIRC cohort and in the CC-e.3 subtype (Figure 4C). Based on 1000 Genome Project data, the minor alleles of both variants (T) were correlated, and therefore one increased the risk of progression (rs1344555-T, Figure 4A) while the other was associated with a relatively higher level of expression of *EVI1* (rs11718241-T, Figure 4C).

The rs16853722 variant did not show significant associations, while analyses of rs75316749 raised the possibility of associations with metastatic ccRCC OS, although the number of informative cases was too small for them to be statistically significant. Five rs75316749 heterozygous (AG) individuals were identified in the metastatic ccRCC cohort, with an estimated HR of 0.30 (log-rank *p =* 0.039; Figure 4C). In this setting, the minor allele G, which has previously been linked to increased lung and breast cancer risk [36], may be associated with a lower probability of death.

## 3. Discussion

This study proposes that evaluation of EVI1 protein or gene expression, and of specific *EVI1* genetic variants, may help improve estimates of prognosis and of benefit from everolimus-based therapy in ccRCC. The connection with cancer progression is supported by immunohistochemical studies of tumors with advanced disease features, and by analyses of gene expression profiles in primary tumors from TCGA. The results are coherent with, and expand on, the proposed oncogenic role of EVI1 in other solid cancers [5,6,7,8,9,10,11,12,13]. However, its impact in ccRCC may be limited to tumors corresponding to the CC-e.3 subtype.

The influence of common genetic variation on everolimus-based therapy is based on the results of variants previously associated with lung function [34,35] and with pleiotropy, including cancer susceptibility [36]. Thus, the depicted genetic variants could also be relevant for predicting progression and/or therapeutic response in other cancer settings. Consistent with the observed genetic associations in metastatic ccRCC, ectopic overexpression of oncogenic EVI1^Del190–515^ confers resistance to everolimus of RCC cell lines. In addition, the resistant phenotype is associated with enhanced pS6 in two models, which builds on previous AKT-mTOR signaling observations in breast and colorectal cancer cells [6,7], and with leukemia [19].

The oncogenic transcriptional program mediated by EVI1 in ccRCC and the potential differential role of *EVI1* isoforms remain to be determined. We may speculate that oncogenic EVI1 in ccRCC is linked to the acquisition of stem cell-like and/or EMT features, as described in other cancer types [5,6,7,8,9,10]. It is of particular note that EMT has also been associated with resistance to allosteric mTOR inhibition [37,38]. The precise causal variants linked to the observed genetic associations are also unknown. Based on data from other cancer models and from eQTL observations, associated risk alleles might increase *EVI1* expression and thereby enhance its function and activate the corresponding oncogenic transcriptional program [7,8,15]. Collectively, the prognostic and therapeutic predictive associations indicate that targeting EVI1 could improve the cure of advanced ccRCC. This may be accomplished by targeting known interactors of EVI1 involved in epigenetic and transcriptional regulation [17], or by targeting metabolic dependences centered on the creatine kinase pathway [39] and L-asparaginase function [40]. These strategies could be combined with allosteric mTOR inhibition; however, while rapalogs do not benefit all patients and do not always produce durable responses, immunotherapy with checkpoint inhibitors is becoming a major choice for the treatment of ccRCC [41]. Interestingly, simultaneously targeting TGF-β and checkpoint inhibitors confers marked inhibition of tumorigenesis in preclinical models [42,43,44]. Given that oncogenic EVI1 modulates TGF-β signaling [24,25], ccRCC cases with EVI1 over-expression might show differential benefit from immunotherapy, and further studies may warranted to assess the potential benefit of targeting EVI1.

## 4. Materials and Methods

### 4.1. Patients

A cohort of 39 ccRCC cases (Supplementary Table S1) collected at the Cruces University Hospital (Barakaldo, Spain) were analyzed for EVI1 expression by immunohistochemical assays. The cohort comprised 9 women and 30 men, who had a median age at diagnosis of 66 years. Their pathological data included tumor grade, diameter, stage, necrosis, sarcomatoid features, lymph nodes affected (yes/no), existence of metastases (yes/no), months of follow-up, and dead or alive status. The institutional ethics committee approved the study and all patients provided informed consent for the study (CEIC-PI2016096).

The cohort of patients genotyped for selected genetic variants corresponded to a prospective, single-arm phase II study of metastatic RCC (93.1% ccRCC) treated with everolimus [45] (Supplementary Table S3). All enrolled patients received no more than two anti-angiogenic therapies before receiving everolimus. There were 19 women and 39 men, who had a median age at diagnosis of 60 years. Immunohistochemical results of AKT1 positivity were based on the H-score method. The ethics committee of the Military Institute of Medicine (Warsaw, Poland) approved the study and all patients provided informed consent for the study of genetic variants.

### 4.2. Immunohistochemistry Assays

The assays were performed on serial paraffin sections of a tissue microarray, applying a protocol including heat-induced epitope retrieval (35 min in a pressure cooker), citrate buffer, 1:50 dilution of anti-EVI1 delta 190–515 antibody (Novus Biologicals, Centennial, CO, USA), and Dako liquid DAB (diaminobenzidine) plus substrate chromogen system (Agilent, Santa Clara, CA, USA). In all experiments, analogous samples were processed without incubation with the primary antibody; no immunostaining was observed in any of these assays. Results were scored blind with respect to clinical information. Molecular analyses were carried out as part of the ProCURE research program, at the Catalan Institute of Oncology, IDIBELL (Barcelona, Spain), and followed the reporting recommendations for tumor marker prognostic studies [46].

### 4.3. Genetic Analyses

DNA was extracted from primary tumors. Samples were lysed using the PrepFiler™ buffer, and substrate was removed using LySep columns. The lysates were loaded onto an AutoMate Express instrument for DNA extraction. Quantitative and qualitative assays of the resulting DNA were carried out using Quantifiler Duo on the Applied Biosystems Real-Time PCR 7500 system. Genotyping was performed using TaqMan assays (Applied Biosystems, Foster City, CA, USA) in ABgene’s Universal Master Mix (Thermo Scientific, Waltham, MA, USA). Replicate samples were assayed, with a template (buffer only) used as negative control. Duplicates consisting of DNA extracted from the same material, but at different times, were analyzed to assess the concordance and quality of the genotyping.

### 4.4. Statistical Analyses

Logistic regressions including age and gender as covariates, and 2 × 2 contingency Fisher’s exact test were used to evaluate immunohistochemical results from tumor and thrombi. The association with survival was assessed by univariate and multivariate (including age, gender, and tumor stage) Cox regression analyses using the survival package in R software. For the genetic studies, the primary end point was PFS, defined as the time elapsed between the date of entry into the study and the date of disease progression or of the most recent follow-up. Secondary end points were OS, the probability of PFS for at least six months, objective response rate, and toxicity, determined by adverse events and laboratory measures. Statistical analyses were performed using STATA (10.0 STATA Corp.) and R software.

### 4.5. TCGA Data Analyses

Gene expression, genomic copy number, genotype, and clinical data were obtained from TCGA (data access #11689) and from the corresponding publications [30,47]. The genotype data corresponded to TCGA results using the Affymetrix Genome-Wide Human SNP Array 6.0 (SNP6). The GSEA tool was used with default parameters in the v4.0.3 Java desktop application [48], and the pre-ranked input corresponded to the Pearson’s correlation coefficients computed between *EVI1* and any other gene analyzed by RNAseq (FPKM log2 scaled).

### 4.6. Cellular Assays

The 786-O, A498 and ACHN cell lines were cultured under standard conditions and confirmed to be free of *Mycoplasma* contamination. *EVI1* full-length and Del190–515 were ectopically overexpressed using a pEGFP-C1 construct [15]. Cells were transfected with GFP or GFP-EVI1-encoding vectors, sorted for GFP positivity at 24 hours and then plated for viability assays. The viability studies were based on colorimetric assays using a tetrazolium compound (MTT) and included exposure to different concentrations of everolimus (Selleck Chemicals, Houston, TX, USA). 

### 4.7. Western Blot and Antibodies

To analyze extracts, cells were lysed in RIPA buffer, lysates were clarified twice by centrifugation, and protein concentrations were measured using the Bradford method (Bio-Rad, Hercules, CA, USA). Lysates were resolved in SDS-PAGE electrophoresis gels and transferred to Immobilon-P (Merck Millipore, Burlington, MA, USA) or PVDF membranes (Sigma-Aldrich, St. Luis, MO, USA). Target proteins were identified by detection of horseradish peroxidase-labeled antibody complexes with chemiluminescence using the ECL Western Blotting Detection Kit (GE Healthcare, Chicago, IL, USA). The antibodies were anti-total and anti-phospho-Ser473 AKT1 (#9272 and #9271, respectively, Cell Signaling Technology, Danvers, MA, USA), anti-total and anti-phospho-Ser235/236-ribosomal S6 protein (#SC-74459, Santa Cruz Biotechnology, Dallas, TX, USA; and #4858, Cell Signaling Technology, respectively), and anti-VCL (V9131, Sigma-Aldrich).

## 5. Conclusions

High levels of expression of *EVI1* in ccRCC are associated with features of cancer progression and invasion, and with poor patient outcome in the CC-e.3 subtype. Common genetic variants in *EVI1* are associated with the response to everolimus of metastatic ccRCC. Determination of EVI1 protein or gene expression, and of defined *EVI1* genetic variants could improve estimates of ccRCC patient outcome and benefit from everolimus in the clinical scenario.

## Supplementary Materials

The following are available online: https://www.mdpi.com/2072-6694/12/2/300/s1. Table S1, Clinical and histopathological features of the retrospective ccRCC cohort used for immunohistochemical studies of EVI1; Table S2, Curated gene sets (GSEA MSigDB C2 set) positively co-expressed with *EVI1* in CC.e-3 tumors; and Table S3, Main clinical and histopathological characteristics of metastatic patients treated with everolimus, used for genetic analyses. Figure S1, The whole western blots.
