# Supplementary material for: EVI1 as a Prognostic and Predictive Biomarker of Clear Cell Renal Cell Carcinoma
Source: Cancers (Basel). 2020 Jan 28;12(2):300. doi: 10.3390/cancers12020300 (PMC7072453; doi:10.3390/cancers12020300)
Supplement: Supplementary file 1 [file cancers-12-00300-s001.zip › cancers-660730-supplement-final/cancers-660737-Supplementary Figure.pdf]

Palomero et al., Fig. 3B (left panels)

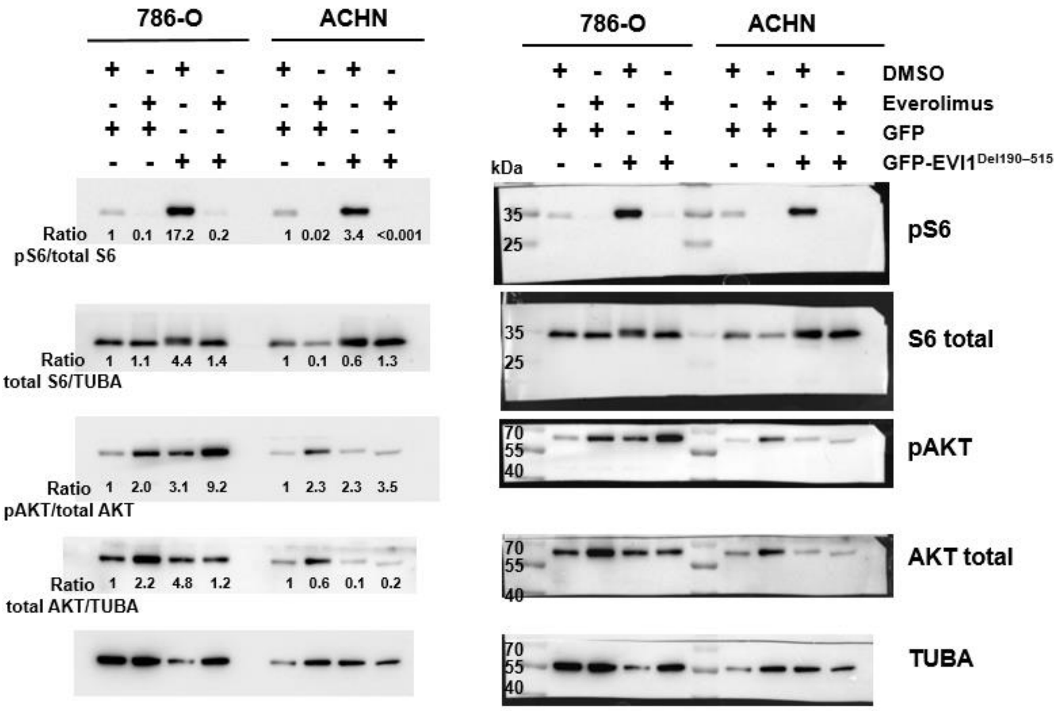

Palomero et al., Fig. 3B (right panels)

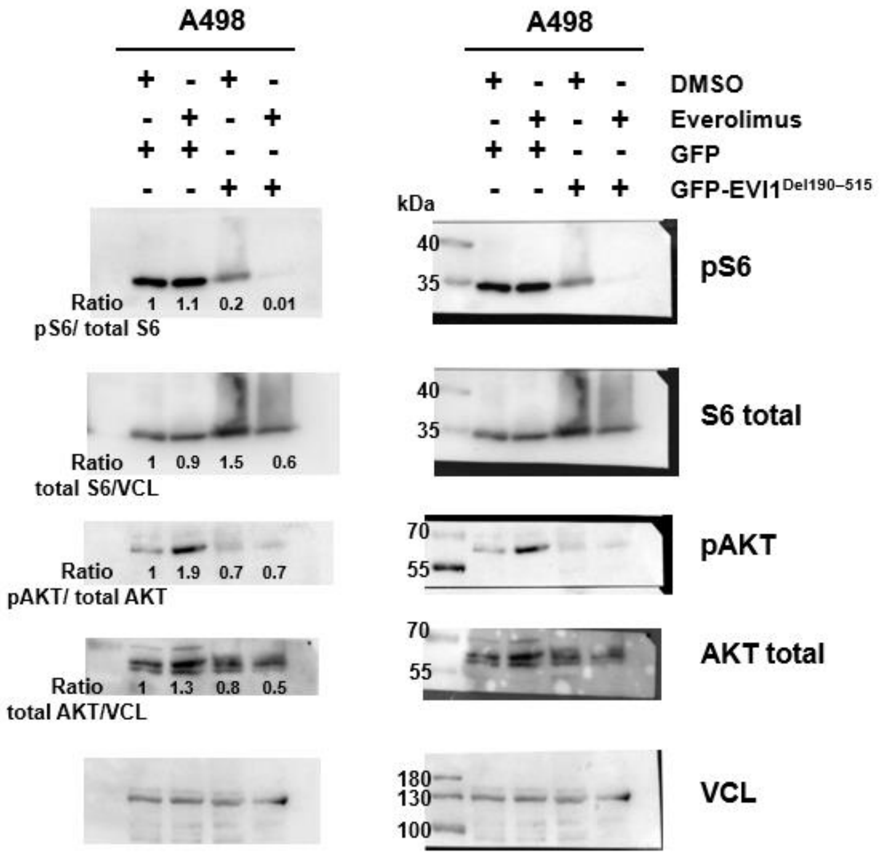

Figure S1. The Whole western blots.
